# Microencapsulation Enhances the Biological Potential, Bioaccessibility, and Intracellular Oxidative Status of Guava Phenolic Extracts

**DOI:** 10.3390/antiox14111334

**Published:** 2025-11-05

**Authors:** Renan Danielski, Sarika Kumari, Pavan Kumar Kakumani, Fereidoon Shahidi

**Affiliations:** Department of Biochemistry, Memorial University of Newfoundland, St. John’s, NL A1C 5S7, Canada; renan.danielski@lethpolytech.ca (R.D.); pkkakumani@mun.ca (P.K.K.)

**Keywords:** natural enzyme inhibitors, cellular antioxidant activity, in vitro digestion, atherosclerosis, antimutagenic

## Abstract

Guava fruit is widely consumed in tropical countries and beyond. The phenolic fraction of guava pulp and processing waste (a single fraction containing seeds, skins, and pulp residues) have been reported to carry in vitro biological activities, acting on biomarkers of metabolic diseases such as type 2 diabetes and obesity (enzymatic inhibition of α-glucosidase and pancreatic lipase), atherosclerosis (mitigation of LDL-cholesterol oxidation), and mutagenesis (suppression of DNA strand scission). However, such bioactivities may be compromised by the exposure of guava phenolics to the harsh conditions found along the human gastrointestinal (GI) tract. To overcome this limitation, guava phenolic extracts were microencapsulated with maltodextrin through freeze-drying. The effect of crude and microencapsulated extracts on biomarkers of metabolic diseases was compared before and after in vitro simulated GI digestion. Moreover, guava waste extracts were tested for their ability to interfere with the intracellular redox status of Caco-2 and HeLa cells incubated with free radicals. Microencapsulation considerably improved the bioaccessibility of guava phenolics across digestion stages, which reflected on the enhancement of most bioactivities measured, with the exception of pancreatic lipase inhibition (both pulp and waste extracts) and LDL oxidative protection (pulp extract). Meanwhile, microencapsulation accentuated intracellular antioxidant activity in Caco-2 cells induced by guava waste extract whereas a prooxidant effect in HeLa cells was intensified. This highlights the selectivity of the same extract toward different cell lines. Overall, microencapsulation was demonstrated as a promising tool for protecting and even enhancing the nutraceutical power of guava phenolics, reinforcing their relevance in the development of functional foods and nutraceutical products.

## 1. Introduction

Guava is popularly consumed in tropical countries, eaten as a raw fruit as well as a part of industrially processed food products, such as frozen pulp, juice, and jams. This places guava as a culturally and industrially relevant raw material. Guava processing is usually performed with the objective of obtaining the fruit’s pulp, the most sought-after fraction, resulting in the discard of seeds, skins, and residual pulp. The unit operation conducted to isolate guava pulp from the remaining portions is usually performed in a pulper, where the leftover components come out as a single fraction containing seeds, skins, and residual pulp [1]. It is estimated that guava waste corresponds to 30% of the fruit’s total mass, a considerable fraction that is usually disposed in landfills or used in low-value applications such as fertilizer and animal feed [2].

Prototypes for the valorization of guava processing waste have been developed experimentally for multiple applications in pharmaceuticals, nutraceuticals, cosmetics, and functional foods [3,4]. The bioactive composition of this fruit is what accredits it for such applications. Guava fruit is a rich source of lycopene, pectin, and has a rich profile of polyphenols. In fact, the polyphenolic fraction of guava pulp and its processing waste have demonstrated a myriad of biological activities including antioxidant potential and the ability to act on biomarkers of chronic diseases [2,5].

When intended for use in food and health-related purposes, natural substances must keep an acceptable level of bioactivity after gastrointestinal (GI) digestion. In other words, the substance must be released from the matrix, maintain its integrity across the GI tract, and be available for intestinal absorption (bioaccessibility). Commonly, after absorption, natural compounds are metabolized, with active metabolites further traveling through the systemic circulation and reaching the target tissues (bioavailability) in order to perform localized action [6]. Substances combining both high bioaccessibility and bioavailability are desirable for the development of pharmaceuticals, and more recently, nutraceuticals (ingredients taken in a medicinal form for health-promotion). In a previous study [5], powdered samples of guava pulp and processing waste subjected to in vitro simulated GI digestion showed low intestinal bioaccessibility, reduction in antioxidant power, as well as diminished ability to inhibit the enzyme α-glucosidase, following a considerable change in their composition of polyphenols when compared to pre-digested samples. Therefore, valorization of guava fractions for biological activity requires certain approaches to protect and stabilize such activities so they can reach the target tissues retaining their health-promoting potential.

Microencapsulation is a strategy that may be used to protect the phenolic integrity of guava bioactive fractions while maintaining the intended biological response. The technique consists of enveloping a target substance with a wall material compatible with the molecule of interest to protect its chemical identity and promote targeted delivery [7]. In other words, microencapsulation creates a barrier against the pH variations and high enzymatic activity found across the GI tract, factors that can easily degrade bioactive compounds and lead to reduced biological activity. The wall material enveloping the target substance aims at retarding its premature release during the GI digestion process, ensuring that the substance reaches the intestinal tract relatively intact for absorption into the enterocytes [8]. Microencapsulation is highly used in the pharmaceutical field and for certain food ingredients. As the nutraceutical area starts to gain traction, microencapsulation has the potential to become a crucial tool for optimizing the use of biologically active molecules [9].

The use of human cell lines to evaluate the biological response of polyphenols and other bioactive compounds is being popularized in recent years [10]. Established models such as Caco-2 (human colorectal adenocarcinoma cells) monolayer for studying intestinal absorption and HeLa (cervical cancer) cells for assessing anticarcinogenic properties have higher physiological relevance than other in vitro models. Cell lines can mimic certain scenarios of the human physiology, providing mechanistic insights into the effects of molecules of interest. Data obtained from cell models are complementary to chemical and biochemical assays and serve to lay the foundation for future animal studies and clinical trials. Similarly to encapsulation techniques, using human cell models in bioactive research is a helpful tool to confer a solid scientific and technological basis for the expansion of the nutraceutical area.

The microencapsulation of phenolic extracts obtained from guava fruit (edible and discarded portions) and the impact of this technique on the bioactivities, digestibility, and intracellular effects have not so far been investigated. As such, it was hypothesized that microencapsulating guava crude extracts will protect phenolic structural integrity, reflecting on the conservation of biological properties and improving the bioaccessibility of these target molecules. Therefore, this study aimed to microencapsulate phenolic extracts obtained from guava pulp and processing waste powders using a simple, untargeted approach. Specifically, it intended to investigate the behavior of these microencapsulated guava phenolic extracts under in vitro simulated GI digestion and assess their biological relevance by evaluating their capacity to mitigate oxidative damage in two different cell models.

## 2. Material and Methods

### 2.1. Material

The chemicals 2,2-diphenyl-1-picrylhydrazyl (DPPH), 2,2′-azinobis(3-ethylbenzothiazoline-6-sulfonic acid) (ABTS), 2,2′-azobis(2-methylpropanimidamide dihydrochloride) (AAPH), 2,4,6-tris(2-pyridyl)-S-triazine (TPTZ), *p*-nitrophenyl glucopyranoside, 4-nitrophenyl-octanoate, human low-density lipoprotein (LDL), supercoiled plasmid pBR322 DNA, phenolic standards, bile salt, and enzymes α-glucosidase from *Saccharomyces cerevisiae* (≥10 units/mg of protein), lipase from porcine pancreas (100–500 units/mg of protein), 2′,7′-dichlorodihydrofluorescein diacetate (DCFH-DA, ≥97% purity), 2,2′-azobis(2-amidinopropane) dihydrochloride (ABAP), 3-(4,5-dimethylthiazol-2-yl)-2,5-diphenyltetrazolium bromide (MTT), Dulbecco’s Modified Eagle Medium (DMEM), and fetal bovine serum (heat inactivated) were purchased from Thermo Fisher Scientific (Nepean, ON, Canada). The enzymes α-amylase, pepsin, pancreatin, and Viscozyme-L, dimethyl sulfoxide (DMSO), L-glutamine, penicillium-streptomycin solution (10,000 units), and trypsin-EDTA solution (0.25%), as well as maltodextrin of high dextrose equivalent (DE 16.5–19.5), Folin–Ciocalteu’s phenol reagent and other solvents and chemicals of analytical and chromatographic grade were obtained from Sigma-Aldrich Ltd. (Oakville, ON, Canada). Human colon adenocarcinoma (Caco-2) and cervical carcinoma (HeLa) cells were obtained from ATCC (American Type Cell Culture, Manassas, VA, USA).

### 2.2. Methods

The experimental approach used in the present study consisted of three parts:

1. Production of microencapsulated extracts and initial characterization: The soluble phenolic fraction from guava pulp and waste powders was obtained and microencapsulated. The microencapsulated extracts were characterized for their physicochemical properties, total phenolic and flavonoid contents, antiradical capacity and ferric reducing ability, α-glucosidase and pancreatic lipase inhibition, oxidative protection to LDL-cholesterol and supercoiled DNA, and phenolic profile by HPLC-UV-MS-TOF. Results were compared with unencapsulated extracts obtained from the same sources.
2. Simulated gastrointestinal (GI) digestion of microencapsulated extracts: Microencapsulated extracts and their unencapsulated counterparts were subjected to an in vitro GI model comprising oral, gastric, small intestine, and large intestine stages. The phenolic bioaccessibility (%) was assessed by comparing the total phenolics released from microencapsulated extracts during each digestion phase with the output of their unencapsulated counterparts. The antiradical and ferric reducing capacity were also measured at each digestion stage. Finally, the bioaccessible fraction (small intestine digesta) of each extract was assessed for α-glucosidase and pancreatic lipase inhibition, as well as oxidative protection to LDL-cholesterol and supercoiled DNA.
3. Impact of microencapsulated extract on the redox status of human cell lines: The microencapsulated extract showing the best performance on the previous parts was chosen for testing in Caco-2 and HeLa cells. The extract was incubated with each cell line at multiple concentrations for a short period and evaluated for cytotoxicity (MTT assay) and cellular antioxidant activity (CAA).

#### 2.2.1. Preparation of Guava Powders

Guava powders were produced from fresh red guavas (*Psidium guajava* L. ‘Pedro Sato’ variety) procured from a market in Florianópolis, SC, Brazil. Guava fruits were loaded into a pilot-scale pulper (Brameitar, Campinas, SP, Brazil), where the pulp was separated from the residual waste fraction (a single fraction containing seeds, skins, and pulp leftovers). The pulp and waste fractions were dried in a forced-air oven at 55 °C for 5 h (TE-394/2 Tecnal, Piracicaba, SP, Brazil) and ground in a hammermill (TE-090 Tecnal, Piracicaba, SP, Brazil) to fine powders with particle sizes of 0.500–0.550 mm and were stored at −20 °C until used for the extraction of soluble phenolics.

#### 2.2.2. Extraction of Soluble Phenolic Compounds

Prior to the extraction of soluble phenolics, guava pulp and waste powders were defatted with hexane (1:5, *w*/*v*). The defatted powders (2 g each) were then combined with 30 mL of 30% (*v*/*v*) ethanol and sonicated in an ultrasonic water bath (300 Ultrasonik, 155 W, Ranch Cucamonga, CA, USA) for 20 min at room temperature. The supernatant was collected, and the solid residue was re-extracted two additional times. The combined supernatants were taken to a rotary evaporator (R-300, Büchi, Flawil, Switzerland) at 40 °C for ethanol removal. Crude extracts were frozen at −40 °C for 48 h and freeze-dried (Freezone 6, Labconco, Kansas City, MO, USA) for 72 h. Freeze-dried phenolic extracts were ground with a mortar and pestle. A fraction of each powdered extract was collected for microencapsulation while the remaining was analyzed as unencapsulated extracts for comparison purposes.

#### 2.2.3. Microencapsulation of Phenolic Extracts

The microencapsulation of freeze-dried phenolic extracts from guava pulp and waste was based on the protocols of Ballesteros et al. [11] and Pashazadeh et al. [12] in combination with preliminary tests. A 20% maltodextrin (dextrose equivalent 16.5–19.5) aqueous solution was prepared, in which 2.5 g of freeze-dried phenolic extract was dispersed. The solution was homogenized with a Polytron (PT 3000, Brinkman Instruments, Rexdale, ON, Canada) at 8000 rpm for 20 min. Subsequently, the solution was frozen at −40 °C for 48h and freeze-dried (−48 °C, pressure at 0.01 mbar, single cycle) for 72 h. The dried microencapsulated extracts were ground in a coffee bean grinder into fine powders. The payload was calculated according to Equation (1).

(1) $$Payload\;\left(\%\right)=\left\lfloor \left(\frac{mass\;(g)\;of\;crude\;phenolic\;extract}{mass\;(g)\;of\;resultant\;microencapsulated\;extract\;}\right)\right\rfloor \times 100$$

#### 2.2.4. Physicochemical Characterization of Microencapsulated Extracts

##### Moisture Content

The moisture content (%) of microencapsulated extracts was determined by oven drying 5 g of sample at 105 °C (about 4 h). Samples were cooled in a desiccator until reaching constant weight. The mass difference between the pre-dried and post-dried powder was used to calculate the moisture content [13].

##### Hygroscopicity

This determination followed the protocol by Kuck and Noreña [14]. Microencapsulated extracts were weighed (2 g) in Petri dishes, and placed in a desiccator containing a saturated solution of sodium sulfate (around 80% of relative humidity) for one week at 25 °C. Then, samples were weighed again to account for the moisture gain, which was expressed as g of moisture per 100 g of dry solids (g/100 g).

##### Particle Size and Polydispersity Index

The average particle size of microencapsulated extracts, as well as polydispersity index (PDI) were measured by dynamic light scattering (DLS) using a zetasizer (Nano ZS, Malvern Instruments Ltd., Worcestershire, UK). Firstly, the extracts were dispersed in deionized water (1:100, *w*/*v*) under magnetic agitation. The dispersed extracts were then placed in the appropriate cuvettes for the DSL measurements at 25 °C in three successive determinations for each sample.

#### 2.2.5. Bioactive Characterization of Microencapsulated Extracts

Microencapsulated and unencapsulated guava extracts were dissolved in methanol to yield a concentration of 5 mg/mL for analyses involving bioactive characterization and biochemical assays.

##### Total Phenolic and Total Flavonoid Content

The total phenolic content (TPC) of microencapsulated and unencapsulated guava extracts (before and after simulated digestion) was determined according to Singleton and Rossi [15], with modifications. In a 96-well microplate, diluted Folin–Ciocalteu phenol reagent consisting of phosphomolybdic acid and tungstate (25 μL), samples (25 μL), and ultrapure water (200 μL) were combined and reacted for 5 min. Then, 10% sodium carbonate (25 μL) was added and the reaction continued for 1 h in the dark at room temperature. Microplates were read in a microplate reader (BioTek Synergy Mx microplate reader, BioTek Instruments, Inc., Winooski, VT, USA) at 725 nm. Results were calculated according to a gallic acid standard curve (0–135 mg/mL) and reported as mg of gallic acid equivalents (GAE) per g of dried extract (mg GAE/g).

Total flavonoid content (TFC) was determined according to Kim et al. [16], with modifications. Diluted samples (1 mL) were placed in a Falcon tube along with 5% sodium nitrite (0.3 mL) and reacted for 5 min. Then, 10% aluminum chloride (0.3 mL) was included and after 1 min, 1 M sodium hydroxide (2 mL) was also added. Following centrifugation at 4000× *g* for 5 min, the supernatant was collected and allowed to react for 15 min in the dark at room temperature. The absorbance was recorded in a UV-visible spectrophotometer (HP8452 A diode array spectrophotometer, Agilent Technologies, Palo Alto, CA, USA) at 510 nm. Results were calculated based on a catechin standard curve (0–100 mg/L) and expressed as mg of catechin equivalents (CE) per gram of dried extract (mg CE/g).

Determination of core and surface phenolics

The core and surface phenolics of each microencapsulated extract were measured in order to calculate the encapsulation efficiency. For core phenolics, a total of 100 mg of encapsulated extract was dissolved in 1 mL of ethanol/acetic acid/water (50:8:42, *v*/*v*/*v*) and the mixture was vortexed for 1 min [17], centrifuged at 4000× *g* for 10 min, with the supernatant being analyzed for TPC as per Section Total Phenolic and Total Flavonoid Content. Surface phenolics were determined by dissolving 100 mg of encapsulated extract into 1 mL of ethanol/methanol (1:1, *v*/*v*) solution for 1 min [17]. The mixture was centrifuged at 4000× *g* for 10 min and the supernatant was analyzed for TPC, according to Section Total Phenolic and Total Flavonoid Content. The encapsulation efficiency (%) was calculated by Equation (2).

(2) $$Encapsulation\;efficiency\;(\%)=\left[\left(\frac{{TPC}_{Core\;Phenolics}-{TPC}_{Surface\;Phenolics}}{{TPC}_{Unencapsulated\;extract}}\right)\right]\times 100$$

##### Antioxidant Activity

2,2-Diphenyl-1-picrylhydrazyl (DPPH) scavenging capacity

The DPPH antiradical activity of microencapsulated and unencapsulated guava extracts was determined according to Kitts et al. [18], with modifications reported by Lima et al. [19]. An aliquot of 260 μL of a 0.10 mM DPPH methanolic solution was added to a microplate along with 40 μL of sample, reacting in the dark for 30 min at room temperature. Absorbance was read at 515 nm using a microplate reader. The blank consisted of methanol in place of guava extracts. Results were calculated by using a Trolox standard curve (0–100 ppm) and reported as micromolar Trolox equivalents per gram of dry extract (μmol TE/g).

2,2′-Azinobis-(3-ethylbenzothiazoline-6-sulfonic acid) (ABTS) scavenging capacity

This determination followed the protocol of Nenadis et al. [20], modified by Rufino et al. [21]. The ABTS radical cation solution was prepared by mixing 140 mM potassium persulfate (88 μL) with 7 mM ABTS stock solution (5 mL). The radical solution (290 μL) was mixed with 10 μL of extract in a microplate, with the reaction taking place in the dark for 6 min at room temperature. The absorbance was read in a microplate reader at 734 nm. The blank consisted of ethanol in place of guava extracts. Results were calculated by using an ascorbic acid standard curve (30–280 mg/L) and reported as micromolar of ascorbic acid equivalents per gram of dry extract (µmol AAE/g).

Ferric reducing antioxidant power (FRAP)

This determination was performed according to Benzie and Strain [22], with modifications. FRAP reagent was prepared at the time of the analysis by mixing 10 mM 2,4,6-Tris(2-pyridyl)-s-triazine (TPTZ) diluted in 40 mM HCl, sodium acetate buffer, and 20 mmol/L iron chloride (III) hexahydrate. Samples (20 μL) and FRAP reagent (280 μL) were mixed in a microplate and incubated at 37 °C for 30 min (MaxQ 4000, Thermo Scientific, Waltham, MA, USA). Then, the absorbance was read in a microplate reader at 593 nm. The blank contained methanol in substitution to the extracts. Results were calculated by an ascorbic acid standard curve (15–150 mg/L) and reported as micromolar of ascorbic acid equivalents per gram of dry extract (µmol AAE/g).

##### Biochemical Assays

α-Glucosidase and pancreatic lipase inhibitory activity

Microencapsulated and unencapsulated extracts (diluted in buffer solution to hold a concentration of 5 mg/mL) were tested for enzymatic inhibition capacity (α-glucosidase and pancreatic lipase), according to Danielski and Shahidi [2]. An aliquot of 5 μL of α-glucosidase (10 U/mL) was mixed with 0.1 M PBS (pH 6.8, 620 μL) and 10 μL of extract in an Eppendorf tube. In parallel, pancreatic lipase diluted in Tris-HCl buffer (5 mg/mL, 100 μL) was mixed with 4 mL of 1 M Tris-HCl acetate buffer (pH 8.5) and 100 μL of extract in a test tube. Both reaction mixtures were incubated at 37 °C for 25 min, followed by the addition of the substrate (10 μL of *p*-nitrophenyl glucopyranoside for α-glucosidase and 100 μL of 5 mM 4-nitrophenyl octanoate for pancreatic lipase) and further incubation for 25 min. Sample blanks were prepared in the same manner without enzyme addition, while controls did not contain any sample. The reaction was terminated by either adding 650 μL of 1 M sodium carbonate (α-glucosidase) or by immersing test tubes into an ice bath (pancreatic lipase). The absorbance of the yellow-colored product (*p*-nitrophenyl) was read at 410 nm for α-glucosidase inhibition and at 412 nm for lipase inhibition using a UV-visible spectrophotometer. The % α-glucosidase and pancreatic lipase inhibition was calculated by Equation (3).

(3) $$\%\;Enzyme\;Inhibition=\left[1-\left(\frac{{Abs}_{Sample}\;-\;{Abs}_{Sample\;Blank}}{{Abs}_{Control}\;-\;{Abs}_{Control\;Blank}}\right)\right]$$

Inhibition of cupric ion-induced human low-density lipoprotein (LDL) peroxidation

This determination was performed according to Ambigaipalan and Shahidi [23]. Prior to the experiment, human LDL-cholesterol (5 mg/mL) was dialyzed for 12 h at 4 °C in a dialysis tube of molecular weight cut-off of 12–14 kDa under nitrogen atmosphere in the absence of light. Then, the molecule was diluted 100 times in 10 mM PBS (containing 0.15 M NaCl, pH 7.4). Diluted LDL (800 μL) was placed in an Eppendorf tube with 100 μL of extract (diluted in PBS buffer), being initially incubated at 37 °C for 15 min. Next, the reaction was initiated by adding 100 μM cupric sulfate (100 μL) and the mixture was incubated for up 22 h. Absorbance (234 nm) was monitored at 2 h intervals in a UV-Vis spectrophotometer as a measure of conjugated dienes (CD) generation. Sample blanks did not contain LDL nor cupric sulfate. The LDL blank did not contain samples nor cupric sulfate, while the negative control was devoid of samples. CD suppression by guava extracts was calculated according to Equation (4).

(4) $$Suppression\;of\;CD\;formation\;\left(\%\right)=\left[\left(\frac{{Abs}_{Negative\;control}\;-\;({Abs}_{Sample}\;-\;{Abs}_{Sample\;Blank}}{{Abs}_{Negative\;Control}\;-\;{Abs}_{LDL\;Blank}}\right)\right]\times 100$$

Inhibition of hydroxyl radical-induced supercoiled DNA strand scission

This determination followed the protocol reported by Ambigaipalan and Shahidi [23]. A hydroxyl solution was prepared by combining 0.5 mM FeSO_4_ (5 μL) with 1 mM H_2_O_2_ (5 μL). The radical (10 μL) was mixed with 5 μL of extract, 5 μL of 0.5 mM PBS (pH 7.4), and 5 μL of supercoiled pBR322 plasmid DNA (50 μL), being incubated for 1 h at 37 °C. Subsequently, a 0.7% agarose gel was prepared and added of 10 μL of SYBR safe stain. After incubation, 5 μL of loading dye composed of bromophenol blue (0.25%), xylene cyanol (0.25%), and glycerol (50%) was included in the reaction mixture, which was loaded onto the agarose gel (aliquots of 10 μL). Next, electrophoresis was performed at 80 V for 2 h in an electrophoresis chamber (model B1A, Owl Separation Systems Inc., Portsmonth, NH, USA) filled with Tris-acetate-EDTA (pH 8.5) buffer and connected to a power supply (model 300 V, VWR International Inc., West Chester, PA, USA). A blank (containing DNA and PBS) and a negative control (containing DNA, PBS, and hydroxyl radical solution) were run concomitantly. DNA bands were detected in the Bio-Rad ChemiDoc image system (Bio-Rad Laboratories, Hercules, CA, USA), and the area of bands was determined. The retention of supercoiled DNA (%) was calculated by Equation (5).

(5) $$\%\;Supercoiled\;DNA\;retention=\left[\left(\frac{Area\;of\;supercoiled\;DNA\;with\;radical\;and\;sample}{Area\;of\;supercoiled\;DNA\;(blank)}\right)\right]\times 100$$

##### Identification and Quantification of Phenolic Compounds by High Performance Liquid Chromatography with Ultraviolet Detection Coupled with Time-of-Flight Mass Spectrometry (HPLC-UV-MS-TOF)

Phenolic compounds contained in microencapsulated and unencapsulated guava extracts were identified and quantified according to Danielski and Shahidi [5]. Microencapsulated extracts were pre-digested in order to release their phenolic content by dissolving in a mixture of ethanol/acetic acid/water (50:8:42, *v*/*v*/*v*), followed by vortexing and centrifugation at 4000× *g* for 10 min. The extract was dried in a rotary evaporator and resuspended in HPLC-grade methanol. Unencapsulated extracts were dissolved in HPLC-grade methanol to yield the same concentration (5 mg/mL). The HPLC Agilent 1260 system was equipped with a quaternary pump (G1311A), a degasser (G1379A), an ALS automatic sampler (G1329A), an ALS Therm (G1130B), a Colcom column compartment (G1316), a diode array detector (DAD, G4212B), and a system controller connected to a Chem Station Data handling system (Agilent Technologies, Palo Alto, CA, USA). The system was coupled with an Agilent 6230 TOF system (TOF LC/MS) with electrospray ionization (ESI), operating in negative mode. MS parameters were at scan range from *m*/*z* 100 to 2000, drying nitrogen gas at 350 °C, flow of 12 L/min, and nebulizer gas pressure of 70 psi. The Agilent LC-MSD software (version 2.8) was used for data analysis.

Compound separation occurred through a Synergi Fusion LC-18 column (50 × 2 mm, 4 μm, Phenomenex) with a binary mobile phase consisting of 0.1% formic acid in water (mobile phase A) and 100% methanol (mobile phase B) at a flow rate of 0.150 mL/min. Elution gradient was as follows: 0 min–90% A; 5 min–10% A; 7 min–10% A; 10–17 min–90% A; afterward mobile phase A was increased to 100% at 17 min, followed by column equilibration from 17 to 22 min. Before injection (10 μL), the samples were filtered using a 0.45 μm PTFE membrane syringe filter (Thermo Scientific, Rockwood, TN, USA). Phenolic acids and flavonoids were detected at 280 nm. Phenolic acids (protocatechuic, *p*-coumaric, 4-hydroxybenzoic, *trans*-cinnamic, syringic, sinapic, gallic, caffeic, ferulic, and ellagic acids, flavonoids ((+)-catechin, (−)-epicatechin, and quercetin), and the stilbene resveratrol were identified by comparing their retention times and ion fragmentation patterns with authentic standards under the same operational conditions. Other compounds were tentatively identified using tandem mass spectrometry (MS^n^) and literature data. The quantification of such compounds was based on their corresponding aglycones, and the results were reported as µg/g of sample.

#### 2.2.6. In Vitro Simulated Gastrointestinal (GI) Digestion

Microencapsulated and crude guava extracts in powder form were subjected to in vitro simulated GI digestion, according to the protocol described by Danielski and Shahidi [5]. Digestion consisted of 1 g of each sample submitted to sequential (1) oral, (2) gastric, (3) small intestine, and (4) large intestine digestive stages. Each digestion buffer was standardized to 10 mL and the protocol was performed as follows:

1. Oral: One gram of extract was mixed with 75 U/mL α-amylase and 0.75 mM calcium chloride dissolved in 0.01 M PBS (pH 7.4). The pH was adjusted to 6.5 with a pH meter (FisherBrand, AB315, Waltham, MA, USA) using 5M HCl and 5M NaOH solutions. The mixture was incubated at 37 °C for 10 min under constant stirring (200 rpm). Incubation conditions (except for time) was the same for all digestion stages.
2. Gastric: The orally digested pellet was combined with 2000 U/mL pepsin and 0.075 M calcium chloride diluted in PBS. The pH was adjusted to 2 and incubation lasted for 2 h.
3. Small intestine: The gastric-digested pellet was mixed with 100 U/mL pancreatin and 10 mM bile salt containing 0.3 mM calcium chloride dissolved in PBS. The pH was adjusted to 7.4 and incubation lasted for 3 h.
4. Large intestine: The intestinally digested pellet was combined with 30 μL of Viscozyme-L enzyme blend (cellulase, hemicellulose, arabanase, β-glucanase, and xylanase) in PBS. The pH was adjusted to 4 and incubation lasted for 16 h.

After each stage, the supernatant was collected and immersed into an ice bath to cease enzymatic activity. Then, digestion extracts were centrifuged (Thermo Scientific Sorvall LYNX 6000 Superspeeid centrifuge, Thermo Fisher Scientific, Pittsburgh, PA, USA) at 4000× *g* for 10 min and stored at 4 °C for further analysis within 2 days.

#### 2.2.7. Cell Culture and Sample Dilution

Both the Caco-2 and HeLa cells were cultivated in a similar manner, according to the protocol described by Danielski et al. [24]. Cell culture media were composed of Advanced DMEM supplemented with 10% fetal bovine serum (FBS, endotoxin-free, heat-inactivated), 1% L-glutamine, and 1% penicillium-streptomycin solution. Cells were thawed and seeded in Corning 25 cm^2^ culture flasks, being later transferred to 75 cm^2^ flasks upon reaching confluency (over 80%, confirmed by microscopic observation). Cells were kept incubated under humidified atmosphere with 5% CO_2_ at 37 °C. Caco-2 cells were used at passage numbers 36–40, while HeLa cells were used at passage numbers 3–7. For cell-based assays, microencapsulated and unencapsulated guava extracts were diluted in cell culture medium to yield concentrations of 2.5, 50, 100, 150, 200, and 250 μg/mL. Dilutions were prepared on the day of the assays and used to determine cell viability and intracellular oxidative status, following the methodologies described by Kellet et al. [25].

#### 2.2.8. Cell Viability

Cell viability was measured by the 3-(4,5-dimethylthiazol-2-yl)-2,5-diphenyltetrazolium bromide (MTT) assay. Cells were plated in Corning Costar^®^ 96-well, black, flat bottom tissue culture-treated dishes at a density of 1 x 10^6^ cells/well and incubated at 37 °C for 24 h in order to reach 100% confluency. Next, the cells were aspirated and washed with 0.01 M PBS (pH 7.4) thrice, followed by the addition of 50 μL of culture medium and 50 μL of diluted sample (triplicate wells for each concentration). A negative control was prepared with 100 μL of culture medium instead of samples. The plate was incubated at 37 °C for 1 h, followed by aspiration and PBS washing. Then, the wells were filled with culture medium (100 μL) and 12 mM MTT (10 μL) and incubation followed for 4 h. Subsequently, a medium aliquot was removed (85 μL) from each well and 50 μL of dimethyl sulfoxide was added. An additional incubation period of 10 min preceded absorbance reading of the formazan complex formed at 540 nm in a microplate reader (BioTek Synergy Mx microplate reader, BioTek Instruments, Inc., Winooski, VT, USA). Sample absorbance values that were significantly (*p* < 0.05) lower than the absorbance of the control were considered cytotoxic, as only live cells can produce formazan. The relative cell viability (%) of was calculated according to Equation (6).

(6) $$Relative\;cell\;viability\;\left(\%\right)=\left(\frac{{Abs}_{Treated\;cell}}{{Abs}_{Control\;cell}}\right)\times 100$$

#### 2.2.9. Intracellular Oxidative Status

Cells were plated as described in Section Cell Viability. After the PBS wash, 50 μL of 25 μM DCFH-DA working solution (10 μL in 5 mL of culture medium) was added to the wells, followed by 50 μL of diluted extracts (triplicate wells for each concentration). A negative control was prepared by replacing the extracts with culture medium. The plate was incubated for 1 h at 37 °C, followed by media aspiration and PBS wash. Then, each well received 100 μL of 600 μM ABAP (free radical solution) and the fluorescence was immediately read (excitation at 485 nm and emission at 538 nm) in a microplate reader using the kinetic function, in which readings were automatically taken every 5 min for 1 h. Fluorescence decay curves were obtained and the area under the curve (AUC) was calculated by integration and converted to cellular antioxidant activity (CAA) units by using Equation (7).

(7) $$CAA\;unit=\%\;Fluorescence\;decline=\left[1-\left(\frac{{AUC}_{Treated\;Cells}}{{AUC}_{Control\;Cells}}\right)\right]\times 100$$

#### 2.2.10. Statistical Analyses

For statistical analysis, IBM SPSS 27.0 for MacOS (SPSS Inc., Chicago, IL, USA) was employed. Results were analyzed by one-way analysis of variance (ANOVA), where significant differences in the mean values of dependent variables across the samples were evaluated. When significant differences were detected, means were compared by Tukey’s HSD test (*p* < 0.05). For pairwise comparisons, the Bonferroni test (*p* < 0.05) was used. MTT and intracellular oxidative status assays were performed thrice. Results were reported as mean ± standard deviation (SD) of triplicate measurements.

## 3. Results and Discussion

### 3.1. Physicochemical Characterization of Microencapsulated Extracts

Soluble phenolic extracts were obtained from guava pulp and processing waste powders and microencapsulated by freeze-drying, employing maltodextrin (20% in solution) as a wall material. The physicochemical characterization of microencapsulated extracts is displayed in Table 1.

The moisture content of guava pulp microencapsulated extract (10.1 g/100 g) was higher than its corresponding processing waste extract (8.9 g/100 g). These results are similar to those reported in the literature on microencapsulated fruit extracts produced by freeze-drying. Kuck and Noreña [14] reported a moisture content of 7.65–8.06 g/100 g of powdered sample for microencapsulated phenolic extract of grape skin. According to the authors, microcapsules obtained by freeze-drying usually display a higher moisture content than those produced by spray-drying, which can be due to the small pores of the microcapsules’ outer layer formed by the rapid freezing process. This may hinder mass transfer during the sublimation process, thus increasing water retention.

The hygroscopicity of microencapsulated extracts was 15.3–16.2% (Table 1), also in agreement with other encapsulated extracts reported in the literature, such as jaboticaba bark extract using guar gum and maltodextrin as wall material (17.75%) and Bordo grape pigment using maltodextrin as the sole encapsulating material (12.4-17.4%), as reported by Silva et al. [26] and de Souza et al. [27], respectively. In samples obtained from juice and fruit extracts, hygroscopicity is generally due to low molecular weight carbohydrates and organic acids with low glass transition temperature.

The payload indicates the amount (g) of active ingredient contained in the microcapsules, which can be measured by dividing the mass of phenolic extracts used for the encapsulation process by the mass of resultant microcapsules. Several factors are known to affect this parameter, including the solubility of the target substance in the wall material, the method and conditions of encapsulation, molecular weight of target compounds, and encapsulation efficiency [28]. In general, achieving payloads over 25% is considered desirable for microencapsulation of bioactive compounds, which was attained in both microencapsulated samples (51% for microencapsulated pulp extract and 26% for microencapsulated waste extract). The observed variability among samples may be due to their different phenolic composition, leading to lower or higher interaction between the compounds present and the wall material, as well as the ability to stay entrapped in the microcapsule’s core.

The encapsulation efficiency, based on the TPC of extracts before and after encapsulation was 89.5% for guava pulp extract and 79.3% for guava waste extract. The combination of maltodextrin as an encapsulating agent and the freeze-drying technique may have contributed to the high encapsulation efficiency. Maltodextrin of high dextrose equivalence (DE) has been used due to its low permeability to oxygen and elevated stability, which factors in the protection of antioxidant substances, such as polyphenols. Coupled with that, freeze-drying is an appropriate dehydration method for heat-sensitive compounds, offering better protection and bioactivity retention than drying techniques involving high temperatures for prolonged times [29].

Both microencapsulated extracts presented particle sizes < 500 µm, confirming their classification as microcapsules (Table 1). This feature favors the applicability of such extracts in a wide range of products. As discussed by Comunian et al. [30], about 1/3 of the food produced worldwide is wasted throughout the processing chain. One solution for reincorporating these portions into the food chain is by the extraction and further microencapsulation of their bioactive compounds, which can serve as health-promoting natural ingredients in functional foods and nutraceuticals.

The polydispersity index (PDI) of guava waste extract (0.733) was considerably higher than that of guava pulp extract (0.134). Lower PDIs are often desirable since they suggest that microcapsules in the sample have uniform and similar sizes, positively impacting product performance and controlled released of the encapsulated bioactives. Although this characteristic is not crucial for most food products, it can be a determinant for other applications such as drug delivery systems and cosmetics, among others [31].

### 3.2. Estimation of Total Phenolic Content (TPC) and Total Flavonoid Content (TFC)

**Figure 1:**
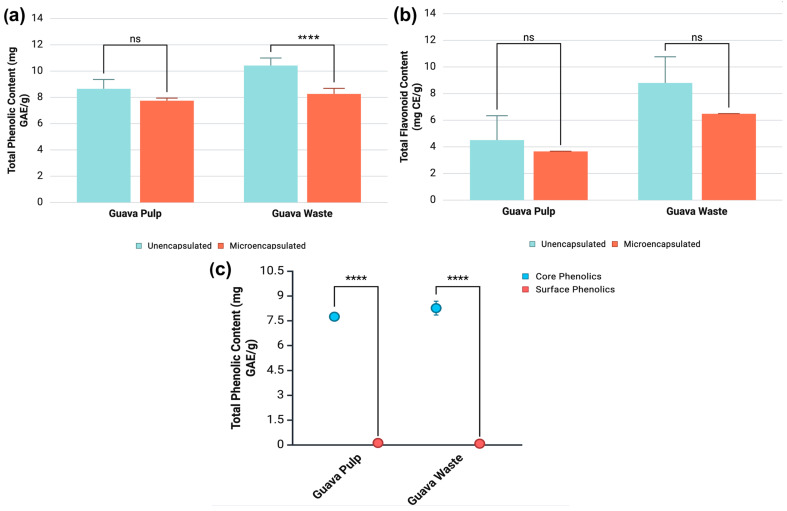
Results for (**a**) total phenolic content, (**b**) total flavonoid content, and (**c**) core and surface phenolics of unencapsulated and microencapsulated extracts obtained from guava pulp and processing waste. Results reported as mean ± standard deviation (SD). **** Indicates significant difference (*p* < 0.05) by the Bonferroni test between unencapsulated and microencapsulated samples. ns = no significance.

The total phenolic and flavonoid contents of unencapsulated and microencapsulated guava phenolic extracts are compared in Figure 1. The TPC of guava pulp extract did not vary significantly between its unencapsulated and microencapsulated versions (7.75–8.66 mg of gallic acid equivalents (GAE)/g) On the other hand, guava waste extract exhibited a loss in total phenolics upon the microencapsulation procedure, representing a decline of approximately 20%.

As shown in Figure 1c, phenolics were mostly found in the core of the microcapsules, which was indicated by the very low content of surface phenolics (0.082–0.124 mg GAE/g) detected both in guava pulp and waste microencapsulated extracts. This result attests to the effectiveness of the microencapsulation protocol employed since the objective of encapsulation procedures is to entrap target substances into the core of the wall material network. High contents of surface phenolics in encapsulated extracts can lead to oxidation and degradation, contradicting the primary objective of encapsulation technologies. The TFC (Figure 1b) was constant across unencapsulated and microencapsulated extracts, without any significant flavonoid losses due to the encapsulation procedure. Overall, guava waste extracts (6.48–8.80 mg of catechin equivalents (CE)/g) demonstrated higher TFCs than guava pulp extracts (3.66–4.51 mg CE/g).

### 3.3. Phenolic Composition

The phenolic composition of guava’s pulp and waste microencapsulated extracts is shown in Table 2.

A total of eight phenolic compounds were identified in the microencapsulated guava pulp extract, including five phenolic acids, two flavonoids, and one ellagitannin. Ferulic, *trans*-cinnamic, and sinapic acids, as well as *p*-coumaroyl molonyldihexoside, (+)-catechin, and pinocembrin were below the limit of detection in the crude extracts. Conversely, in the microencapsulated form, these compounds displayed significant amounts (11.11–44.95 μg/g), with *p*-coumaroyl malonyldihexoside as the major polyphenol (44.95 μg/g). Meanwhile, ellagic acid and ellagic acid derivative were contained in both crude and microencapsulated extracts. Ellagic acid in the microencapsulated extract retained 52% of its original content, while its derivative form was enriched in the microencapsulated extract (29.61 μg/g), being the second-highest quantified phenolic in this sample.

The sum of the content of each individual phenolic quantified by HPLC-UV-MS-TOF revealed that guava pulp’s microencapsulated extract accounted for a total phenolic content of 175.5 μg/g, which is significantly lower than the amount of soluble phenolics in the original extract (437.7 μg/g) quantified by the same technique. Analyzing the shift in the polyphenolic profile upon microencapsulation, it is possible to notice that several ellagitannins (ellagic derivative III, ellagic acid pentoside, and ellagic acid hexoside) and vanillic acid (aglycone and glycoside forms) contained in the crude extract did not migrate to the microencapsulated extract. As they were major contributors to the soluble phenolic content of the original samples, their loss may explain the reduced phenolic concentration found in the microcapsules. The microencapsulation technique used was selective for simple phenolic acids and flavonoids, with very few polyphenols of higher degree of complexity being detected. These compounds could have been either degraded or broken down into simpler molecules during the encapsulation procedure. Alternatively, these derivatives could have presented limited solubility in the maltodextrin solution, which restricted their loading into the microcapsules’ core. Further studies are needed to clarify the fate of unencapsulated phenolics.

A microencapsulation-induced phenolic profile shift was also observed for guava waste extract (Table 2). A total of ten compounds were identified, including six phenolic acids, two flavonoids, one ellagitannin, and one proanthocyanidin. From those, three were also part of the composition of the crude extract. *p*-Coumaroyl malonyldihexoside was present in trace amounts in guava waste’s crude extract. Following microencapsulation, this compound showed a content of 49.69 μg/g, the second-highest quantified phenolic in this sample, demonstrating that microencapsulation was effective in protecting this *p*-coumaric glycoside, as verified in both guava pulp and waste extracts. However, protocatechuic acid went from 19.49 μg/g in the unencapsulated extract to only trace amounts in the microencapsulated form. *trans*-Cinnamic acid was quantified in higher concentration in the microencapsulated extract (32.45 μg/g) than in the crude extract (23.37 μg/g).

Ellagic acid pentoside, previously detected in the unencapsulated extract, was not present after microencapsulation. Nevertheless, the aglycone form of ellagic acid was detected in the microcapsules and not in the crude extract. It is possible that ellagic acid (aglycone) could have stemmed from the breakdown of its glycosidic form during the microencapsulation process. Besides ellagic acid, quercetin, ferulic acid, epicatechin gallate, 1-*O*-(4-coumaroyl)-glucose, and ellagic acid derivative were also detected in microencapsulated guava waste extract. Ellagic acid derivative was the major compound, with 52.20 μg/g. Meanwhile, B-type proanthocyanidin trimer was below the limit of quantification, probably due to its high molecular weight which could have prevented loading into the microcapsules. This corroborates the fact that the microencapsulation technique used in the present study was selective toward simpler phenolic compounds. Although the polyphenol composition of guava waste extracts varied between the crude and microencapsulated forms, the total phenolic content was very similar (microencapsulated extract—201.80 μg/g, crude extract—210.98 μg/g).

### 3.4. Antiradical and Reducing Activity

The antiradical activity of unencapsulated and microencapsulated guava phenolic extracts was compared using the DPPH and ABTS assays (Table 3). Although guava pulp extract maintained a similar level of total phenolics before and after microencapsulation, its capacity to scavenge DPPH radical was reduced by about half upon microencapsulation (8.53 vs. 17.11 μmol TE/g for the unencapsulated extract). On the other hand, the microencapsulated guava waste extract demonstrated higher DPPH scavenging activity (8.21 μmol TE/g) than its unencapsulated version (6.74 μmol TE/g) despite the sample’s lower TPC. The effect of microencapsulation on both guava extracts highlights the lack of a positive correlation between TPC and DPPH antiradical activity. Chemical-based assays can provide a generalist overview of the antioxidant mechanism of phenolic mixtures, without discriminating the individual contribution of each component. The antioxidant capacity of complex phenolic mixtures is a result of the interplay between the different polyphenols contained in the pool. The synergistic and antagonistic effects stemming from the interaction among phenolics play a central role in the antioxidant activity and other bioactivities of natural extracts. It is possible that the microencapsulated guava waste extract contained phenolics displaying a stronger affinity for DPPH radical, leading to a higher neutralization level. In order to confirm this hypothesis, further studies can analyze the individual contribution of phenolics composing microencapsulated guava waste extract toward DPPH antiradical capacity.

The outcome of the ABTS assay led to a similar observation. The scavenging of ABTS radical cation was approximately 20% lower for the microencapsulated guava pulp extract in contrast to the crude version. Meanwhile, the same parameter was improved from 75.51 μmol AAE/g in crude guava waste extract to 82.48 μmol AAE/g after the microencapsulation procedure. However, this trend was reversed when analyzing the ferric reducing antioxidant power (FRAP). Stronger FRAP was observed for guava pulp extracts after microencapsulation, contrary to guava waste extract in which the activity was diminished in almost 10% in relation to its unencapsulated counterpart. In general, microencapsulation weakened the antiradical capacity of guava pulp extracts, while enhancing the sample’s ability to reduce Fe^3+^ to Fe^2+^. Contrarily, microencapsulation enhanced the radical scavenging power of guava waste extract while diminishing its ferric reducing ability.

The superior antiradical activity displayed by microencapsulated guava waste extract as opposed to microencapsulated guava pulp extract could potentially be related to the presence of flavonoids (quercetin and epicatechin gallate) in the former. Quercetin is one of the model flavonoids when it comes to achieving maximum antioxidant activity. Its chemical structure combines all the features reported in the literature as ideal for achieving effective radical scavenging, such as the presence of OH groups at C3 and C4 (ortho configuration) on the B ring; a double bond between C2 and C3, presence of a 4-oxo group, and OH group at C3 on the C ring; OH group at C5 on the A ring [32]. Meanwhile, epicatechin gallate is a glycosylated flavonoid, meaning that its C ring lacks some of the structural features contained in quercetin aglycone, theoretically resulting in lower antioxidant capacity. However, the combination of both quercetin and epicatechin gallate could have favored the antiradical power of guava waste extract in the microencapsulated form. These flavonoids were absent from microencapsulated guava pulp extract, as well as from the unencapsulated extracts from both fractions of the fruit.

### 3.5. Effect of Microencapsulated Guava Extracts on Biomarkers of Metabolic Diseases

#### 3.5.1. Inhibition of Metabolic Enzymes

α-Glucosidase is a hydrolase that acts on the α-1,4 glycosidic bonds of disaccharides and oligosaccharides during the digestive process of carbohydrates. This enzyme is one of many that work to release glucose units for intestinal absorption, serving as a model to study pharmaceuticals intended for type 2 diabetes management. Substances capable of inhibiting the enzymatic activity of α-glucosidase consequently hamper glucose release into the bloodstream, preventing postprandial blood sugar spikes [33]. Ideally, these substances should not fully prevent carbohydrate digestion since the fermentation of undigested polysaccharides in the colonic environment leads to side effects related to abdominal discomfort, negatively impacting the quality of life of diabetic patients. Recent literature suggests that the inhibitory mechanism of dietary phenolics toward α-glucosidase does not fully impair the enzyme. It rather slows down the digestive process of polysaccharides, resulting in a progressive release of glucose into the bloodstream, avoiding hyperglycemic peaks [34].

**Figure 2:**
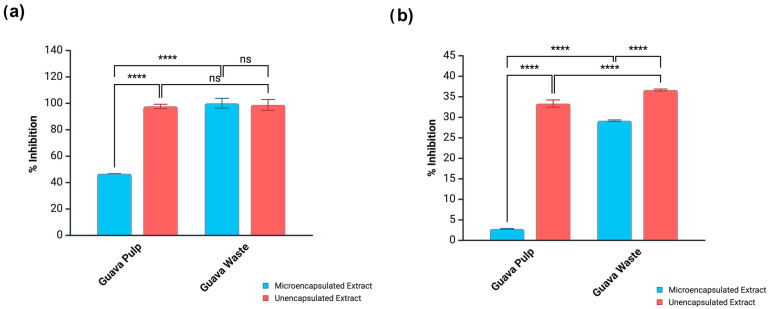
Enzyme inhibitory activity of unencapsulated and microencapsulated guava extracts toward (**a**) α-glucosidase and (**b**) pancreatic lipase Results reported as mean ± standard deviation (SD). **** Indicates significant difference (*p* < 0.05) by the Bonferroni test between crude and microencapsulated samples. ns = no significance.

Crude guava pulp extract at a concentration of 5 mg/mL promoted a high α-glucosidase inhibition rate (97.70%), as seen in Figure 2. At the same concentration, its microencapsulated counterpart reduced the inhibition rate by more than half (46.75%), which could have been prompted by the drastic reduction in the phenolic level after microencapsulation (from 437.7 to 175.5 μg/g, as quantified by HPLC). This effect was not observed when testing guava waste extract as a natural α-glucosidase inhibitor. Both crude (98.86%) and microencapsulated (100%) extracts were equally effective in hampering enzyme activity, which is consistent with the minimal variation in the TPC (201.8–210.98 μg/g, as quantified by HPLC) before and after the microencapsulation of guava waste extract. In fact, a Pearson correlation analysis (*p* < 0.05) showed moderate and positive correlations between α-glucosidase inhibition and TPC (0.5695), TFC (0.6049), total phenolics quantified by HPLC (0.4730), and ABTS scavenging activity (0.6782). Ali et al. [35] also found that TPC, TFC, and ABTS positively correlated with α-glucosidase inhibition promoted by phenolic extracts. Therefore, these parameters might be able to predict phenolic performance as α-glucosidase inhibitors, although correlation alone does not imply causation.

Interestingly, ferulic acid, sinapic acid, and *p*-coumaroyl malonyldihexoside were negatively correlated with α-glucosidase inhibition (−0.4929, −0.9921, and −0.5306, respectively). A study conducted by Aleixandre et al. [34] demonstrated that simple phenolic acids with methoxy substitutions in their structures, such as ferulic and sinapic acids, were less effective at inhibiting α-glucosidase than unsubstituted phenolic acids, including those with more than one hydroxyl group in the chemical structure. According to the authors, these substitutions can introduce a steric barrier to inserting the phenolic into the enzyme’s active site, diminishing the phenolic-enzyme affinity, crucial for the inhibitory event. In the present study, the stronger negative correlation between sinapic acid and α-glucosidase inhibition could be related to the fact that sinapic acid contains two methoxy substitutions as opposed to ferulic acid with only one.

Pancreatic lipase is an enzyme that breaks down triacylglycerols into free fatty acids and monoacylglycerols during fat digestion, being essential for lipid absorption and metabolism [36]. Thus, this enzyme is a target for obesity treatment. Orlistat is the only drug approved by the Food and Drug Administration (FDA) that acts as a pancreatic lipase inhibitor. Nevertheless, this medication has been extensively reported as causing gastrointestinal discomfort and other side effects when taken regularly [37]. Similarly to α-glucosidase inhibitors, there is a quest for alternative therapies targeting pancreatic lipase, with polyphenols suggested as promising candidates. In the present study, microencapsulation drastically reduced the lipase inhibitory activity of guava pulp extract from 33.35 (crude extract) to 2.79% (microencapsulated extract), similar to what was observed for the same sample in the α-glucosidase assay (Figure 2). A reduction in lipase inhibition levels was also observed for microencapsulated guava waste extract, although at a lesser extent. Crude guava waste extract reduced lipase activity by 36.66%, while its microencapsulated counterpart caused a reduction of 29.17%.

Lipase inhibition was also negatively correlated with ferulic acid (−0.4483), sinapic acid (−0.9787), and *p*-coumaroyl malonyldihexoside (−0.4852), while being positively correlated with protocatechuic acid (0.4782). Past studies [37] have found that procyanidins and other oligomeric polyphenols can efficiently act as pancreatic lipase inhibitors while simple phenolics such as monomeric catechins and phenolic acids showed minimal impact. This observation may explain the reduction in lipase inhibitory activity of microencapsulated extracts since they are predominantly composed of simple phenolic acids. Martinez-Gonzalez et al. [38] demonstrated through a molecular docking analysis that phenolic acids and flavonoids tend to bind pancreatic lipase close to the active site, mainly driven by hydrogen bonding and π-stacking interactions. As such, molecules with steric hindrance and methoxy substitutions (lower hydrogen bonding capacity) usually display a compromised ability to inhibit pancreatic lipase. Meanwhile, phenolics with more than one hydroxyl group in their structure, such as protocatechuic acid, have an increased likelihood of hydrogen bonding close to the enzyme’s active site, promoting inhibition. This may explain the positive correlation between the presence of protocatechuic acid in guava extracts and pancreatic lipase inhibition.

#### 3.5.2. Suppression of Oxidative Damage to LDL-Cholesterol and Supercoiled DNA

**Figure 3:**
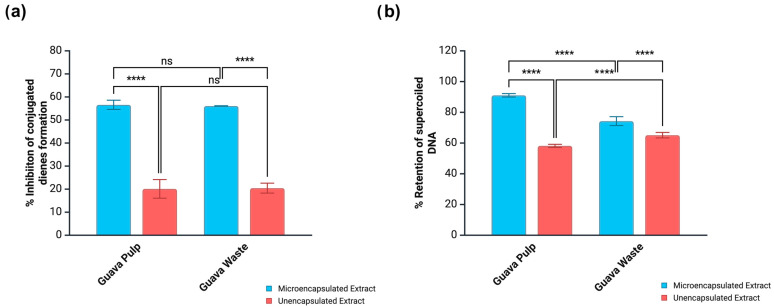
Biological activity of crude and microencapsulated guava extracts measured as (**a**) mitigation of cupric ion-induced conjugated diene formation from LDL-c and (**b**) retention of supercoiled DNA incubated with hydroxyl radical. Results reported as mean ± standard deviation (SD). **** Indicates significant difference (*p* < 0.05) by the Bonferroni test between crude and microencapsulated samples. ns = no significance.

Perturbations to the cellular redox balance resulting in ROS overproduction is a common denominator in the development of chronic inflammation and a myriad of related diseases. For instance, oxidative damage to LDL-cholesterol leads to the generation of a toxic species that is eventually engulfed by macrophages. This is the triggering event involved in the formation of atherosclerotic plaques [39]. At same time, the literature also suggests a relationship between cellar redox imbalance and cancer development. In this case, ROS accumulation is related to a manifold effect, resulting in cellular adaptation, higher proliferation rates, DNA mutations, and genome instability. Cancer cells can adapt to elevated ROS levels, allowing them to survive at concentrations that would be cytotoxic to healthy cells. In addition, prolonged periods of oxidative distress can cause the desensitization of DNA repair mechanisms, and some common radical-induced deformations include DNA base modifications (e.g., 8-hydroxyguanine), as well as single- and double-strand breaks [39]. Figure 3 shows the ability of microencapsulated and unencapsulated guava extracts to mitigate cupric ion-induced LDL-cholesterol oxidation and hydroxyl radical-induced strand breaks on supercoiled DNA.

Microencapsulation greatly improved the capacity of both guava pulp and waste extracts to protect LDL-c and supercoiled DNA. While the formation of conjugated dienes (primary oxidation products) from LDL-c was suppressed by 20.1–20.4% by crude extracts, the protection level was increased to 56.1–56.6% when the same extracts were microencapsulated (Figure 3). These results were positively correlated (*p* < 0.05) with the TPC obtained by HPLC quantification (0.4734), as well as with the presence of sinapic (0.5832) and ellagic (0.6612) acids in the extracts. Microencapsulation was also beneficial to the capacity of guava extracts to retain DNA in the supercoiled form when under attack of hydroxyl radicals, from 58.2 to 65.1% (crude extracts) to 74.3–91.1% (microencapsulated extracts). These results also showed a strong and positive correlation with sinapic acid (0.8802), which is relevant to explain the performance of microencapsulated guava pulp extract since this was the only sample containing this phenolic acid.

### 3.6. Impact of Microencapsulation on the Phenolic Bioaccessibility of Guava Extracts

**Figure 4:**
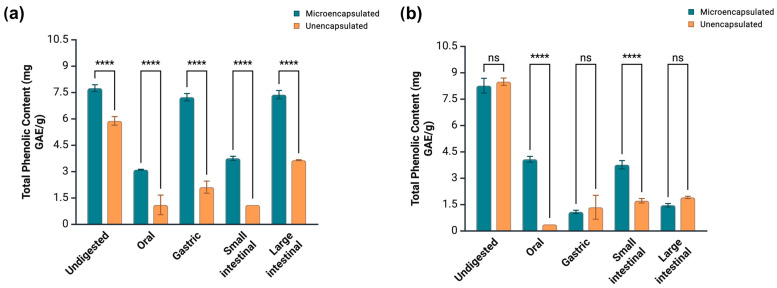
Total phenolic content of crude and microencapsulated (**a**) guava pulp and (**b**) waste extracts upon in vitro simulated GI digestion. Results reported as mean ± standard deviation (SD). **** Indicates significant difference (*p* < 0.05) by the Bonferroni test between crude and microencapsulated samples. ns = no significance.

Crude and microencapsulated guava extracts were subjected to a sequential in vitro digestion model. At each stage, total phenolics and antioxidant capacity were measured, while the enzymatic inhibition and suppression of LDL-c and supercoiled DNA oxidative damage were measured in the bioaccessible fraction (corresponds to the small intestine stage). TPC for crude guava pulp extract varied from 1.10 to 3.65 mg GAE/g across digestion phases. The largest phenolic release happened at the large intestine stage, with the remaining stages showing no significant difference in the TPC level (Figure 4a). Meanwhile, microencapsulated guava pulp extract presented higher TPC across all digestion stages (3.11–7.24 mg GAE/g), in which the gastric and large intestinal phases were responsible for the most significant releases, followed by the small intestinal and oral phases (Figure 4a). Therefore, microencapsulation ensured an increased phenolic concentration during the entire simulated digestion process for this extract.

Meanwhile, TPC for guava waste extract at gastric and large intestinal phases did not vary significantly between the crude and microencapsulated versions of this sample. Nevertheless, microencapsulation increased phenolic release at the small intestinal phase from 1.72 (crude extract) to 3.77 mg GAE/g (microencapsulated extract). The same was observed after oral digestion, in which crude guava waste extract registered a TPC of 0.377 mg GAE/g, as opposed to 4.08 mg GAE/g achieved by its microencapsulated counterpart (Figure 4b). Microencapsulation not only favored phenolic release during simulated GI digestion but also the antiradical and reducing activities of guava extracts. Table 4 shows that both microencapsulated pulp and waste extracts displayed enhanced DPPH and ABTS scavenging activities than their unencapsulated versions at all digestion steps. The same outcome was observed when the FRAP assay was performed. Possibility, the increase in antioxidant capacity was a consequence of the more prominent phenolic concentration in the digested fractions of microencapsulated extracts.

**Figure 5:**
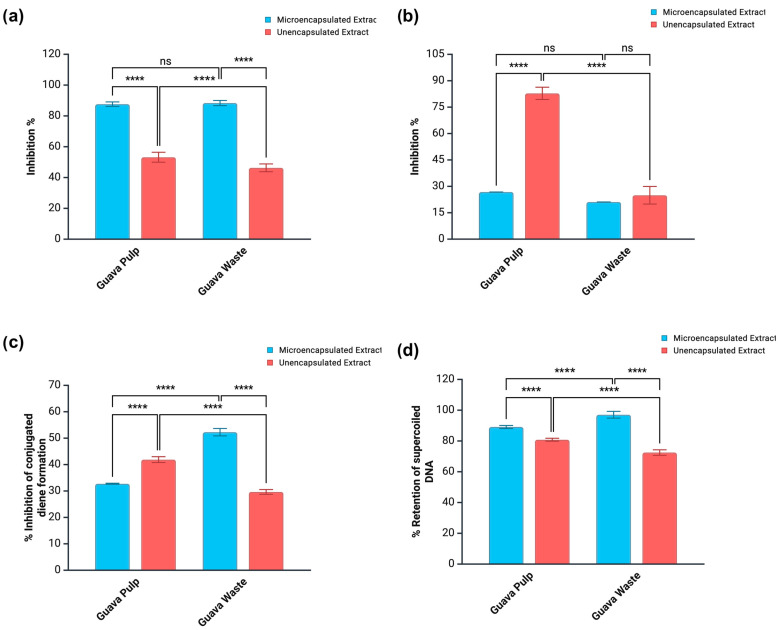
Biological activity of crude and microencapsulated guava pulp and waste extracts upon small intestine digestion, measured as (**a**) α-glucosidase inhibition, (**b**) pancreatic lipase inhibition, (**c**) mitigation of cupric ion-induced conjugated diene formation from LDL-c, and (**d**) retention of supercoiled DNA incubated with hydroxyl radical. Results reported as mean ± standard deviation (SD). **** Indicates significant difference (*p* < 0.05) by the Bonferroni test between crude and microencapsulated samples. ns = no significance.

The potential of guava extracts to act as α-glucosidase inhibitors after small intestinal digestion was amplified when extracts were in the microencapsulated form (Figure 5a). For guava pulp extract, the inhibition level grew from 53.2 (crude form) to 87.6% (microencapsulated form), while for guava waste extract, the change was from 43.3 (crude form) to 88.3% (microencapsulated form). However, the same tendency was not observed for pancreatic lipase inhibition (Figure 5b). Microencapsulated guava waste extract was slightly less effective in inhibiting this enzyme (21.1%) than its unencapsulated counterpart (24.9%). On the other hand, a drastic decline was registered for guava pulp extract. While the digested crude form could inhibit 82.8% of pancreatic lipase’s activity, the microencapsulated version could only inhibit 26.8%.

**Figure 6:**
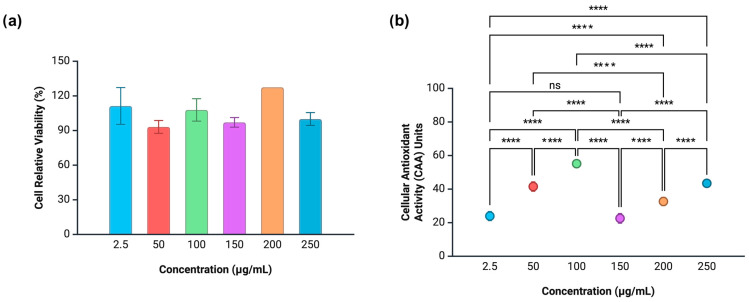
(**a**) Relative cell viability (%) of Caco-2 cells treated with microencapsulated guava waste extract and (**b**) cellular antioxidant activity (CAA). Cells were incubated in 96-well culture plates and treated with the same amount of 25 μM DCFH-DA and extract (2.5, 50, 100, 150, 200, and 250 μg/mL) for 1h. After PBS wash, they were incubated at 600 μM ABAP. Fluorescence was measured for 1 h at the excitation and emission wavelengths of 485 and 538 nm, respectively. CAA units represent cellular antioxidant capacity. Data reported as mean ± standard deviation (n = 3). **** Indicates significant difference (*p* < 0.05) by Tukey’s post hoc test. ns = no significance.

The small intestine fraction of guava pulp extract displayed lower LDL-c oxidative protection when microencapsulated. While the crude extract could inhibit the formation of conjugated dienes by 41.9%, the microencapsulated extract showed an inhibition rate of only 32.7% (Figure 5c). On the other hand, the same bioactivity was favored by microencapsulation in the case of guava waste extract, which increased the inhibition rate from 29.6% in the unencapsulated form to 52.3% in the microencapsulated form (Figure 5d). This discrepancy was not observed when digested extracts were tested for the ability to suppress radical-induced DNA strand break (Figure 6). Microencapsulated pulp and waste extracts showed higher rates of DNA protection (89.1–97%) than their crude versions (72.5–80.8%).

In general, microencapsulation improved the bioaccessibility of phenolic compounds across digestion stages, which reflected enhanced antioxidant activity, oxidative protection to supercoiled DNA, and α-glucosidase inhibition. Nevertheless, pancreatic lipase inhibition was negatively impacted by microencapsulation, especially for guava pulp extract, while LDL-c protection improved for guava waste extract but not for guava pulp extract.

### 3.7. Cytotoxicity and Intracellular Antioxidant Activity

#### 3.7.1. Caco-2 Cells

Since guava waste extract exhibited higher biological potential throughout the study, it was selected for further investigation of its biological relevance. To this end, the microencapsulated extract was incubated with human colorectal adenocarcinoma (Caco-2) cells in order to measure the effect on cytotoxicity and intracellular redox status after a short exposure period (1 h). In parallel, the same experiment was conducted using cervical cancer (HeLa) cells to determine whether the observed effects were cell line specific. The cytotoxicity and intracellular antioxidant activity of the unencapsulated guava waste extract were previously reported by Danielski et al. [24]. In sum, when tested at concentrations ranging from 2.5 to 250 μg/mL, the crude guava waste extract exhibited cytotoxicity at 100 μg/mL, reducing Caco-2 cell viability to 51%. Overall, relative cell viability ranged from 51% to 131%, with the highest viability observed at 50 μg/mL. The cytotoxic effect at 100 μg/mL corresponded to prooxidant activity recorded at the same concentration, whereas all other concentrations demonstrated intracellular antioxidant activity, ranging from 31.4 to 53.6 units of cellular antioxidant activity (CAA). Notably, CAA increased in a concentration-dependent manner from 150 μg/mL to 250 μg/mL, at which point the extract reached its peak antioxidant activity.

Microencapsulation altered the cytotoxicity pattern of guava waste extract, as cytotoxic effects were not observed at any tested concentration (Figure 6a). This indicates that providing a coating layer to polyphenols can alter the extract’s properties, potentially interfering in the cellular mechanisms that involve these substances. It is also important to note that untargeted encapsulation processes can present variances in the extract’s phenolic profile when compared to the crude form, as shown in Table 1. The profile shift can also impact the cytotoxicity and cell viability outcomes of these samples. The relative viability of the microencapsulated extract ranged from 98 to 122% (Figure 6a), showcasing a greater uniformity across concentrations when compared to the unencapsulated form. Cell viability was the highest at 2.5 μg/mL (122%), decreasing to 98% at 50 μg/mL. From this point on, stable cell viability was achieved up to 200 μg/mL, where cell proliferation was observed again (110%).

A stable CAA pattern was achieved upon microencapsulation of guava waste extract (Figure 6b). Antioxidant effects were observed over the entire concentration range tested, with no observed prooxidant effects. CAA increased in a concentration-dependent manner from 2.5 to 100 μg/mL (21.78–54.45 units), followed by a significant drop at 150 μg/mL (25.73 units) and further concentration-dependent increase up to 250 μg/mL (25.73–44.33 units). This sample showed higher Caco-2 oxidative protection at lower concentrations when compared to the higher ones. In addition, while the 100 μg/mL dose induced prooxidant effects when the crude extract was applied, microencapsulation shifted the extract’s behavior by achieving the highest cellular antioxidant power at this same concentration. The effect of microencapsulation on the CAA pattern induced by guava waste extract is in line with the findings of Ferreira-Santos et al. [40] when studying the encapsulation of a pine bark phenolic extract using maltodextrin as a wall material. The authors demonstrated that microencapsulated extracts were more effective at offsetting ROS formation in Caco-2 cells when compared to their unencapsulated counterparts at concentrations lower than 500 μg/mL.

#### 3.7.2. HeLa Cells

Cervical cancer starts in the cells lining the cervix, the lower part of the uterus. It is often caused by persistent infection with high-risk strains of human papillomavirus (HPV). The HeLa cell line, derived from cervical cancer cells, has been crucial in studying various aspects of cancer biology and has contributed significantly to the understanding of cell behavior and response to treatments. HeLa cells have been used in various studies related to the anticancer effects of nutraceutical compounds. Research involving HeLa cells has contributed to the understanding of how certain nutraceutical molecules may influence cancer cell behavior and viability. This information is valuable in the development of potential cancer therapies or preventive strategies.

**Figure 7:**
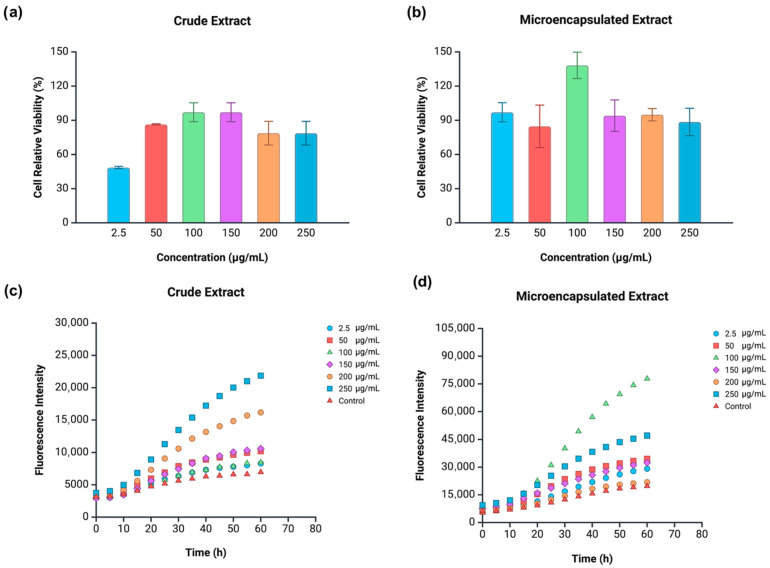
Relative cell viability (%) of HeLa cells treated with (**a**) crude and (**b**) microencapsulated guava waste extracts and fluorescence intensity of HeLa cells treated with (**c**) crude and (**d**) microencapsulated extracts and a free radical source (ABAP). Concentrations producing fluorescence intensity higher than the control (only cells and ABAP) were considered prooxidant. Data reported as mean ± standard deviation (n = 3).

HeLa cells treated with crude guava waste extract followed a concentration-dependent increase in relative viability (up to 100 μg/mL), subsequently showing cell depletion from 150 to 200–250 μg/mL (Figure 7a). The extract displayed cytotoxicity toward HeLa cells under most tested concentrations (48–86% of cell viability), except for the mid concentrations (100 and 150 μg/mL). Around 97% of HeLa cells were viable under non-cytotoxic doses of the extract, and there was no instance where extracts stimulated cell proliferation. Meanwhile, microencapsulation removed guava waste extract’s cytotoxicity towards HeLa cells across all tested dosages (Figure 7b), the same effect produced in Caco-2 cells. In contrast to the crude extract, the microencapsulated guava waste extract exhibited higher levels of cell viability, ranging from 84% (50 μg/mL) to 117% (100 μg/mL). Notably, at the 100 μg/mL concentration, the extract stimulated HeLa cell proliferation, stressing the importance of this threshold for polyphenolic extracts.

Figure 7 shows the progression of ROS generation for each concentration of the phenolic extracts tested in comparison with the control sample, which was devoid of antioxidant molecules. A free radical source (ABAP) was added to all samples prior to fluorescence measure. Treated cells marked by higher fluorescence intensity than control cells indicated stimulation of ROS production in HeLa cells instead of preventing it, characterizing a prooxidant effect. Crude guava waste extract produced prooxidant effects on HeLa cells across the entire concentration range tested (Figure 7c).

Such outcome evidences the extract’s selectivity when it comes to different cell lines. While the sample promoted antioxidative effects on Caco-2 cells under the concentration range studied, it caused oxidative damage to HeLa cells. Oxidative stress can lead to decreased proliferation of cancerous cells by several pathways, from damaging lipids, proteins, and DNA to activating signaling pathways linked to cell apoptosis [41]. As mentioned previously, most concentrations of this extract led to HeLa cell cytotoxicity, except for the 100 and 150 μg/mL. In terms of dose response, higher concentrations of crude guava waste extract (150–250 μg/mL) were able to cause the most significant contribution to ROS proliferation. Microencapsulation of guava waste extract impacted oxidative damage to HeLa cells even further, with an overall higher fluorescence intensity registered when compared to the extract’s crude form (Figure 7d). All tested concentrations caused some level of prooxidative effects, with the 100 μg/mL concentration being the most significant one, followed by 150 μg/mL.

## 4. Cross-Assay Discussion

The present study demonstrated that microencapsulation affected the bioactivity of guava pulp and waste extracts in distinct ways across enzymatic, oxidative, and cellular models. These effects can be primarily attributed to the structural and compositional differences between extracts before and after microencapsulation, as well as to the physicochemical interactions between polyphenols and the encapsulating material. The contrasting order of antioxidant activity (DPPH, ABTS, and FRAP) between pulp and waste extracts further highlights the complexity of phenolic–matrix interactions. These differences could have resulted from the distinct phenolic profile of each fruit portion, a common observation in studies correlating antioxidant activity and phenolic profile [42]. Such differences underscore the influence of phenolic composition and structural diversity on the reaction mechanisms underlying each assay. The interaction of these compounds with encapsulating agents may further modulate their redox reactivity, leading to distinct assay-specific outcomes.

Before digestion, unencapsulated guava extracts exhibited stronger α-glucosidase and lipase inhibitory activities compared with their microencapsulated counterparts. This reduction in inhibitory potential following encapsulation could result from the physical entrapment of phenolic compounds within the maltodextrin network, limiting their immediate access to enzyme binding sites, an effect previously reported in the literature [43]. Conversely, the lack of a barrier hindering the immediate interaction between unencapsulated extracts and enzymes could have favored their inhibitory action. After simulated GI digestion, microencapsulation enhanced the enzyme inhibitory activities of both pulp and waste extracts, indicating that the GI environment favored the controlled release of microencapsulated phenolics. The disintegration of the maltodextrin network under intestinal pH and enzymatic conditions likely increased the access of phenolics to enzymatic binding sites. In contrast, unencapsulated extracts, not being protected by a wall material, might have suffered partial degradation or transformation during digestion, resulting in decreased activity. Therefore, microencapsulation appears to play a protective role across the GI tract, preserving phenolic integrity and enabling their gradual release.

Interestingly, the opposite was observed in redox-related biochemical assays. In both LDL oxidation and DNA strand break models, microencapsulated extracts showed enhanced inhibitory activity compared with their unencapsulated counterparts. This suggests that microencapsulation contributed to the stabilization of redox-active compounds by shielding them from oxidation and polymerization. The protective effect of the maltodextrin barrier appears to maintain the structural integrity and reactivity of key antioxidants, allowing for sustained inhibition of oxidative processes. These findings are in line with previous reports showing that encapsulation improved the stability and bioefficacy of phenolic compounds under oxidative stress conditions [11,40].

Despite the beneficial release effect observed for enzyme inhibition, the antioxidant activity of microencapsulated extracts after digestion showed a moderate decline compared to their pre-digested counterparts. This may be explained by structural transformations of phenolic compounds under digestive conditions, particularly through conjugation, oxidation, or hydrolysis, which can reduce their redox potential. Although microencapsulation delays degradation, prolonged exposure to bile salts and pancreatin can alter the phenolic structure, reducing its overall antioxidant efficiency, as previously noted for polyphenol-rich matrices [40].

The cellular antioxidant activity of guava extracts did not follow a strictly concentration-dependent pattern. This non-linear behavior is frequently observed in cell-based antioxidant assays, reflecting the saturable nature of polyphenol uptake and intracellular metabolism [44]. At higher concentrations, the limited permeability of phenolics or feedback regulation of antioxidant defenses may result in plateau effects. Moreover, the microencapsulated samples exhibited delayed but sustained antioxidant responses, consistent with a slower intracellular release of the active constituents. This inconsistent behavior could also be a result of the short contact time between phenolic extracts and cells (24 h). In some cases, longer incubation times (48 h or more) could be necessary for obtaining a more stable response. Moreover, the distinct fluorescence profiles observed between crude and microencapsulated guava waste extracts also support the influence of release kinetics. In crude extracts, phenolics are readily available to exert immediate antioxidant effects, leading to maximal fluorescence at low concentrations. In contrast, microencapsulated extracts require higher concentrations to achieve similar effects, suggesting a gradual release of phenolics from the encapsulating matrix.

In sum, these findings suggest that the impact of microencapsulation on guava phenolics is dual: it can temporarily limit enzymatic inhibition due to diffusion barriers but enhances compound stability and bioaccessibility under physiological conditions. Therefore, a comprehensive evaluation of microencapsulated fruit bioactives requires integrating pre- and post-digestion assays as well as both chemical and cellular models to fully capture the dynamic interplay between compound stability, release, and bioactivity.

## 5. Conclusions

Phenolic extracts from guava pulp and processing waste were microencapsulated by freeze-drying using maltodextrin as a wall material. The microencapsulated guava pulp extract exhibited higher payload and encapsulation efficiency than its waste counterpart. However, the guava waste extract showed minimal loss of phenolic compounds when compared to its unencapsulated version.

The effectiveness of the microencapsulation process was demonstrated by the predominant localization of phenolics in the core of the microcapsules, with only trace amounts detected on the surface. Additionally, the biochemical effects of microencapsulation varied according to the type of bioactivity and guava extract (pulp or waste). The microencapsulated extracts exhibited increased phenolic bioaccessibility following in vitro simulated digestion while also preserving or enhancing extract protection against LDL-cholesterol and supercoiled DNA oxidative damage. On the other hand, guava pulp extract reduced its effectiveness toward α-glucosidase inhibition upon microencapsulation, with the same effect observed for pancreatic lipase inhibition (in both undigested extracts). When the guava waste extract was incubated with human cell lines (Caco-2 and HeLa), it exhibited a dual effect: an intracellular antioxidant effect in Caco-2 cells and a prooxidant effect in HeLa cells. These responses were intensified by microencapsulation, which also eliminated the cytotoxicity observed at certain concentrations of the unencapsulated extract.

These findings support the potential of microencapsulation to protect and enhance some biological activities of guava extracts, especially those derived from waste, which can be valorized in accordance with circular economy principles. Nevertheless, the post-encapsulation loss of effectiveness observed in some of the biochemical assays performed prompts further adjustments to the microencapsulation protocol used. Moreover, the contribution of non-phenolic guava bioactives, such as lycopene, β-carotene, and tocopherols, to the overall bioactivity of microencapsulated guava extracts merits further investigation. With the necessary adaptations, microencapsulation can represent a step forward in realizing the full potential of guava as a raw material for nutraceutical and functional food applications. It is important to note, however, that these outcomes require validation in animal models and human trials. Future studies should also evaluate the microencapsulated extracts developed in this study using preclinical organisms such as *Caenorhabditis elegans* and fruit fly to gain deeper insights into their metabolization and bioavailability.

## Figures and Tables

**Table 1:** Physicochemical parameters of microencapsulated phenolic extracts of guava pulp and processing waste.

Sample	Moisture (%)	Hygroscopicity (%)	Particle Size (µm)	Polydispersity Index	Encapsulation Efficiency (%) *	Payload (%)
**Guava Pulp**	10.1 ± 0.01 ^a^	16.2 ± 0.5 ^a^	4.0 ± 1.1 ^a^	0.134 ± 0.01 ^b^	89.5 ± 7.1 ^a^	51.0 ± 4.5 ^a^
**Guava Waste**	8.9 ± 1.0 ^b^	15.3 ± 1.6 ^b^	3.1 ± 0.01 ^b^	0.733 ± 0.01 ^a^	79.3 ± 0.4 ^b^	26.0 ± 2.2 ^b^

* Based on the TPC of extracts before and after microencapsulation. Results reported as mean ± standard deviation (SD). Different lowercase letters in the same column represent significant difference (*p* < 0.05) by the *t*-test.

**Table 2:** Identification and quantification (μg/g of powdered sample) of phenolic compounds detected in microencapsulated and unencapsulated guava pulp and waste extracts.

Phenolic Compound	[M-H]^−^ (*m*/*z*)	RT (min)	MS^2^ Ion Fragments	GP-M	GP-U	GW-M	GW-U
***Phenolic acids***							
*trans*-Cinnamic acid ^+^	147	8.6	126, 137	25.01 ± 1.2 ^c^	-	32.45 ± 6.3 ^b^	23.37 ± 2.3 ^a^
Protocatechuic acid ^+^	153	6.8	138	-	-	tr	19.49 ± 2.9 ^b^
Ferulic acid ^+^	193	10.3	133, 173	15.64 ± 0.1 ^d^	-	18.14 ± 5.0 ^d^	-
Sinapic acid ^+^	223	1.067	162	11.11 ± 0.5 ^e^	-	-	-
Ellagic acid ^+^	300	11.2	187, 263	24.11 ± 2.3 ^c^	46.29 ± 4.4 ^a^	26.15 ± 0.9 ^c^	-
1-*O*-(4-Coumaroyl)-glucose	325	7.7	163	-	-	3.773 ± 0.3 ^g^	-
*p*-Coumaroyl malonyldihexoside	411	3.0	137, 205, 251, 375	44.95 ± 6.2 ^a^	-	49.69 ± 7.8 ^a,b^	-
***Flavonoids***							
Pinocembrin	255	11.3	112, 159	-	-	-	-
(+)-Catechin ^+^	289	8.8	245	tr	-	-	-
Quercetin ^+^	301	10.8	113, 127, 137, 139, 159, 183, 217	-	-	4.540 ± 0.7 ^f^	-
Epicatechin gallate	457	6.6	355, 411	-	-	14.82 ± 1.4 ^e^	-
***Ellagitannin***							
Ellagic acid derivative	389	13.6	300	29.61 ± 1.2 ^b^	21.12 ± 1.5 ^b^	52.20 ± 7.7 ^a^	-
***Proanthocyanidin***							
B-type proanthocyanidin trimer	897	12.6	137, 249, 339, 448.3, 554, 746, 840	-	-	tr	-
**Total (μg/g)**				175.5	437.7	201.8	210.98

Abbreviations: GP-M is microencapsulated guava pulp extract; GP-U is unencapsulated guava pulp extract; GW-M is microencapsulated guava waste extract; GW-U is unencapsulated guava waste extract; tr—traces (below the limit of quantification). Results reported as mean ± SD (*n* = 3). Different lowercase letters in the column indicate significant difference according to Tukey’s test (*p* < 0.05). + Positively identified with authentic standards; RT-retention time.

**Table 3:** Antiradical and reducing activity of microencapsulated guava extracts measured by 2,2-diphenyl-1-picrylhydrazyl (DPPH) and 2,2′-azino-bis(3-ethylbenzothiazoline-6-sulfonic acid) (ABTS) scavenging activity and ferric reducing antioxidant power (FRAP), respectively.

Sample	DPPH Scavenging Activity (μmol TE/g)	ABTS Scavenging Activity (μmol AAE/g)	FRAP (μmol AAE/g)
***Guava Pulp***			
Crude extract	17.1 ± 5 ^a^	89.4 ± 1 ^a^	77.0 ± 0.2 ^c^
Microencapsulated extract	8.53 ± 0.5 ^b^	71.6 ± 2 ^c^	98.9 ± 0.8 ^b^
***Guava Waste***			
Crude extract	6.73 ± 0.5 ^c^	75.5 ± 0.4 ^c^	106.6 ± 1 ^a^
Microencapsulated extract	8.21 ± 1 ^b^	82.5 ± 3 ^b^	97.9 ± 0.8 ^b^

Results reported as mean ± standard deviation (SD). Different lowercase letters in the column indicate significant difference (*p* < 0.05) by Tukey’s post hoc test. Abbreviations: TE = Trolox equivalents, AAE: ascorbic acid equivalents.

**Table 4:** Antiradical and reducing activity of microencapsulated guava extracts along in vitro simulated GI digestion, measured by 2,2-diphenyl-1-picrylhydrazyl (DPPH) and 2,2′-azino-bis(3-ethylbenzothiazoline-6-sulfonic acid) (ABTS) scavenging activity and ferric reducing antioxidant power (FRAP), respectively.

Sample	Digestion Stage	DPPH Scavenging Activity (μmol TE/g)	ABTS Scavenging Activity (μmol AAE/g)	FRAP (μmol AAE/g)
**Microencapsulated Guava Pulp Extract**	Oral	27.4 ± 5 ^b^	29.6 ± 0.1 ^d^	10.8 ± 0.2 ^b^
	Gastric	33.1 ± 1 ^a^	40.8 ± 0.1 ^c^	12.2 ± 2 ^b^
	Small intestine	6.69 ± 0.4 ^c^	193.4 ± 3 ^a^	7.28 ± 0.3 ^c^
	Large intestine	25.7 ± 1 ^b^	135.5 ± 2 ^b^	19.0 ± 2 ^a^
**Microencapsulated Guava Waste Extract**	Oral	35.7 ± 0.2 ^a^	31.2 ± 2 ^d^	10.5 ± 0.1 ^b^
	Gastric	33.3 ± 3 ^a^	42.5 ± 0.4 ^c^	11.9 ± 0.2 ^a^
	Small intestine	12.6 ± 3 ^c^	198.7 ± 3 ^a^	11.4 ± 0.4 ^a,b^
	Large intestine	19.4 ± 2 ^b^	131.2 ± 2 ^b^	12.1 ± 0.5 ^a^

Results reported as mean ± standard deviation (SD). Different lowercase letters in the column indicate significant difference (*p* < 0.05) by Tukey’s post hoc test. Abbreviations: TE = Trolox equivalents, AAE: ascorbic acid equivalents.

## Data Availability

The original contributions presented in this study are included in the article. Further inquiries can be directed to the corresponding author.

## Abbreviations

The following abbreviations are used in this manuscript:

ABTS	2,2′-Azino-bis(3-ethylbenzothiazoline-6-sulfonic acid)
AAE	Ascorbic acid equivalent
CE	Catechin equivalent
DPPH	2,2-Diphenyl-1-picrylhydrazyl
FRAP	Ferric reducing antioxidant power
GAE	Gallic acid equivalent
GI	Gastrointestinal
ROS	Reactive oxygen species
TE	Trolox equivalent
TFC	Total flavonoid content
TPC	Total phenolic content
